# Notes

**Published:** 1898-02-26

**Authors:** 


### NOTES.

The Archbishop of Canterbury, as president, took the chair at the annual meeting on February 11th of the Hostel of St. Luke, and reported a deficiency in the receipts, necessitating a call on the reserve fund. Seventy-four patients, representing 43 dioceses, were treated during the year at this nursing home for the clergy.

The Diamond Jubilee Cottage Hospital which was opened on July 20th, 1897, has been moved to the "Town Farm House," which stands alone in a small plot of ground. It has four beds for ordinary cases, two for men and two for women, with one small ward for a private patient. The honorary medical officers are Mr. S. W. Woollett and Mr. A. C. Herbert.

At a public meeting held to consider the proposed Richmond and District Jubilee Cottage Hospital it was stated that £932 16s. 3d. had already been given and promised towards the building. The Marchioness of Zetland, the president of the committee, has offered to give a cottage rent free, as a home for nurse, probationer, &c., and it is hoped sufficient money will be forthcoming to provide a hospital of nine beds for this district.

We understand that the Government have decided to appoint a Royal Commission to inquire as to the best methods of treating sewage. The results said to have been obtained in several places by various methods for the bacterial treatment of sewage, are so remarkable, and seem to suggest the possibility of such radical alterations in our modes of dealing with this vexatious problem, that it is satisfactory to find that an authoritative inquiry is likely to be made on the subject.

The difficulty about appointing a Professor of Surgery at Cambridge has been got over by the recommendation to suspend the professorship and appoint a Reader in Surgery, on whom will fall all the ordinary teaching functions of the professorship. The advice given by the eminent surgeons who were consulted in the matter was to the effect that if the professor appointed were to remain a teacher of any value he ought to have charge of patients at a hospital, and be engaged in the active practice of his profession. This, to all intents and purposes, restricted the choice of the University to one or other of the surgeons of the Addenbrooke's Hospital, and as the hospital appointment was not in the hands of the University, the Readership seemed the only way out of the difficulty. The stipend is to be £240 per annum.

